# Relationship of Social–Emotional Learning, Resilience, Psychological Well-Being, and Depressive Symptoms with Physical Activity in School-Aged Children

**DOI:** 10.3390/children11081032

**Published:** 2024-08-22

**Authors:** Evan Belaire, Fawzi Mualla, Lucas Ball, Iris Ma, Debra Berkey, Weiyun Chen

**Affiliations:** 1 Physical Activity and Health Laboratory, School of Kinesiology, University of Michigan, Ann Arbor, MI 48109, USA; belaire@umich.edu (E.B.); fmualla@umich.edu (F.M.); lukeball@umich.edu (L.B.); irisma@umich.edu (I.M.); 2 Society of Health and Physical Education (SHAPE) Michigan, Lansing, MI 49056, USA; shapemichigandirector@gmail.com

**Keywords:** child health, school programs, physical education, mental well-being

## Abstract

Background: This study investigated the association of psychological well-being (PWB), resilience, depressive symptoms, and social–emotional learning (SEL) with physical activity (PA) in school-aged children. The objective was to understand how these psychosocial factors influence PA levels and identify gender-specific differences in these relationships. Methods: This cross-sectional study involved 534 fourth grade and sixth grade students from eight schools in the Midwest region of the United States, with data collected through a Qualtrics survey. Multiple linear regression models were used to analyze the data, with gender-specific analyses conducted to identify differences between boys and girls. Results: The models indicated that all psychosocial factors taken together are significantly associated with PA (F = 26.937, *p* < 0.001). Of the factors, PWB and resilience were associated with higher PA individually for the total sample (β = 0.383, *p* = 0.001; β = 0.146, *p* = 0.005). A gender-specific analysis revealed that all factors collectively were significantly associated with PA in boys and girls (F = 15.846, *p* < 0.001; F = 6.869, *p* < 0.001). Individually, PWB and resilience were significantly associated with PA in boys (β = 0.358, *p* = 0.001; β = 0.171, *p* = 0.013), while only PWB was significantly associated with PA in girls (β = −0.355, *p* = 0.001). Conclusions: This study highlights the necessity of promoting resilience and psychological well-being through structured physical activities, aiming to reduce the risk of obesity and improve mental health among children. Future research should consider longitudinal designs and objective measures to further elucidate these relationships and inform effective educational strategies.

## 1. Introduction

Physical inactivity among school-aged children has become a critical issue in recent years [1]. According to the Physical Activity Guidelines for Americans [2], children and adolescents aged 6 to 17 years should engage in at least 60 min of moderate to vigorous physical activity (MVPA) each day to promote optimal health and development. Despite the guidelines, a significant number of children fail to meet this daily MVPA requirement, leading to a range of adverse health outcomes [3]. Insufficient physical activity is a major contributor to childhood obesity, which can have long-term negative effects on physical and mental health [4]. The CDC highlights that obesity in children is associated with an increased risk of various health problems, including type 2 diabetes, cardiovascular disease, and psychological issues such as low self-esteem and depression [5].

Schools play a crucial role in providing opportunities for physical activity through physical education classes, recess, and extracurricular sports [6]. Implementing effective physical activity programs in schools can help address the issue of physical inactivity and its associated health problems. By promoting an active lifestyle within the school environment, we can support the long-term health and well-being of children, setting the foundation for a healthier future generation. Therefore, ensuring that children are physically active during school hours is essential for preventing the detrimental consequences of inactivity and fostering a healthier future generation.

Social–emotional learning (SEL) is the process of developing essential skills for understanding and managing emotions, setting and achieving goals, feeling and showing empathy, maintaining positive relationships, and making responsible decisions [7]. SEL encompasses five key domains: self-awareness (recognizing one’s emotions and values), self-management (regulating emotions and behaviors), social awareness (empathizing with others and appreciating diversity), relationship skills (building and maintaining healthy relationships), and responsible decision-making (making ethical and constructive choices) [8]. Research has shown a positive correlation between SEL and physical activity [9]. However, it is important to acknowledge that much of the literature suggests that the relationship between SEL and PA may be bidirectional. For instance, studies indicate that incorporating SEL programs in schools can enhance students’ motivation and participation in physical activities, ultimately leading to better physical health outcomes [10]. Conversely, engaging in regular PA through physical education with incorporated SEL competencies has had effects on PA levels as well. Integrating SEL into physical education not only promotes emotional and social growth but also encourages a more active lifestyle, reducing the risk of obesity and other health issues associated with physical inactivity [11].

Previous studies have demonstrated the positive impact of physical activity on various aspects of students’ lives. One study investigated the effects of a before-school physical activity program on SEL competence among 138 fourth and sixth graders from an elementary and a middle school, with 75 students in the intervention program and 63 in a control group. The results showed a significant improvement in SEL competence among the intervention participants, with a 7–10% increase, whereas the control group showed no change. This highlights the potential benefits of integrating physical activity into school programs to enhance SEL [12]. Moreover, this study suggests that increasing PA in schools could be a viable strategy for improving SEL competencies in children, thereby illustrating the reciprocal nature of the relationship between SEL and PA.

Resilience is the ability of an individual to adapt and recover effectively from stress, adversity, or trauma, maintaining or quickly regaining psychological well-being [13]. A study involving 1732 high school students aged 16 to 20 found that physical activity positively impacts resilience by satisfying three basic psychological needs: competence, autonomy, and relatedness [14]. These needs are essential for human thriving and well-being and serve as sources of nourishment for individuals. Furthermore, additional research underscores the importance of physical activity in building resilience [15,16,17]. Notably, the effects of physical activity on resilience can differ between boys and girls. Research indicates that while physical activity enhances resilience in both genders, boys tend to experience a stronger protective effect from physical activity on mental health resilience compared to girls [18].

Psychological well-being (PWB), defined as a state encompassing emotional stability, positive self-perception, and social functionality [19], is also positively related to physical activity. A review that covered 21 articles with varying interventions with children and adolescents aged between 6 and 18 years explored the relationship between physical education or school sports and psychological well-being. A positive relationship between physical activity, well-being, and other variables, such as basic psychological needs and quality of life, was established based on this review and further literature [20,21,22]. These findings demonstrate the importance of integrating physical activity into the daily lives of children and adolescents to enhance their psychological well-being and overall quality of life.

It is also crucial to consider the specific effects of physical activity on depressive symptoms in children, as these can manifest differently compared to adults. Children may experience depression through persistent sadness, irritability, and a loss of interest in activities, which can affect their social and academic performance. A study found that regular physical activity significantly reduced depressive symptoms in 27 children aged 9 to 11 by improving their mood and overall mental health [23]. This study portrays the importance of incorporating physical activity into children’s routines to enhance their emotional well-being. The findings suggest that even a moderate amount of regular physical activity can significantly benefit children’s mental health [24]. These insights demonstrate the profound impact of physical activity on reducing depressive symptoms in children and emphasize the necessity of integrating regular physical activity into school programs.

Despite the known benefits of physical activity and its correlation with SEL, resilience, psychological well-being, and depressive symptoms, there is a lack of comprehensive studies examining these relationships in the context of school programs. Thus, the purpose of this study is two-fold: (1) to examine the association of SEL, resilience, PWB, and depressive symptoms with physical activity among school-aged students; and (2) to explore gender-specific differences in these relationships. Understanding these dynamics is crucial for developing effective interventions that promote both physical and psychosocial health among students. Therefore, this study proposes two hypotheses: firstly, that all factors taken collectively will have a significant association with physical activity (PA); and secondly, there will be significant differences in the associations between male and female children.

## 2. Materials and Methods

### 2.1. Participants and Study Design

A total of 534 students voluntarily participated in this study, including 415 fourth grade students and 119 sixth grade students, with a mean age of 10 ± 1.018 years. One grade from both the elementary and middle school demographics was selected for the current study, hence the exclusion of the 3rd, 5th, 7th, and 8th grade which are present in the larger study. Participants were recruited from eight schools in rural, urban, and suburban areas across a state in the Midwest region of the United States, to collect baseline data for a larger two-year project. The inclusion criteria were as follows: (1) students were enrolled in the 4th grade or 6th grade at their respective school during the study period; (2) students assented to participate in the study, in addition to parental/guardian approval of the study. Our exclusion criteria included students with physical injuries, mental traumas, mental health/emotional issues, recent hospital stays, or the use of medication that affects mood, behavior, or physical stamina, of which we were informed by the school nurse or social workers.

The participants’ demographic information is presented in Table 1. This study was conducted in accordance with the Declaration of Helsinki, and approved by the Institutional Review Board of the University of Michigan (HUM00241100, approved on 11 May 2023). Written and informed consent was obtained from all participant’s parents/guardians before the study commenced. In addition, all participants assented to participate in the study.

### 2.2. Data Collection

Data for the dependent variable (physical activity levels) and predictor variables (SEL, resilience, psychological well-being, and depressive symptoms) were collected via a Qualtrics survey two weeks before the implementation of the intervention in the larger study. Qualtrics Version January, 2024, is an online tool that allowed the questionnaires to be incorporated into a single survey that the participants could complete electronically. Participants had one week to complete the survey. On the cover page of the Qualtrics survey, participants were instructed to read the assent form, illustrating the purpose of the study, the procedures involved, and the confidentiality of their responses. Prior to proceeding to the survey, participants had options to choose “agree” or “disagree” on the Assent Box. Demographic data were also collected, outlining the frequencies of sexes, grades, ages, and races within the sample. The data collected in this study are all baseline data for a larger two-year project.

#### 2.2.1. Physical Activity

The Modified Physical Activity Questionnaire (Modified PAQ-C) designed by Kolimechkov et al. [25] is a widely used, reliable, and valid measure of general physical activity levels in children during the school year [26] with prior validation in the United States [27]. In the current study, we streamlined the components of the Modified PAQ-C to consist of a single total scale presenting six items that measure the frequency of physical activity participation in a usual week. Using a 5-point Likert scale, the questionnaire assesses participants’ frequency of participation in physical activity during physical education classes, lunch, recess, after school, and during the weekend. Participants were asked to self-rate their participation from 1 (none) to 5 (6 times or more). The scores of the six items are averaged to calculate the final PAQ-C activity score, where a higher score indicates increased physical activity levels. The Modified PAQ-C has a demonstrated moderate internal consistency (α = 0.72) [27].

#### 2.2.2. Social–Emotional Learning

The Student Social–Emotional Learning Questionnaire is a survey designed to assess students’ social–emotional learning skills based on the core competencies and indicators outlined by the Michigan Department of Education [28]. The 15-item questionnaire is comprised of a single total scale with five sub-dimensions. Items 1–3 gauge self-awareness, 4–6 evaluate self-management, 7–9 assess social awareness, 10–12 consider personal relationships, and 13–15 examine decision-making skills. Participants were asked to respond to each item using a 5-point Likert scale, ranging from 1 (not true at all) to 5 (always true). The scores of all 15 items are then averaged to compute a final score ranging from 1, representing low SEL competencies, to 5, signifying high SEL competencies. In this study, the Cronbach’s alpha value of the total scale is 0.932, indicating an acceptable internal consistency.

#### 2.2.3. Psychological Well-Being

The Five Well-Being Index (WHO-5) designed by the World Health Organization [29] consists of a single scale containing 5 positively phrased items scored using a 6-point Likert scale. The WHO-5 is a widely used, valid, and reliable measure of general well-being specifically over the past 2 weeks, applicable to sample populations regardless of underlying illness or conditions [30]. Participants were asked to self-rate their sense of positive well-being from 0 (at no time) to 5 (all the time), with higher ratings reflecting improved well-being. The scores of the 5 items are aggregated, resulting in a raw score ranging from 0 to 25, with 0 indicating the worst possible and 25 the best possible quality of life. The raw score is consequently multiplied by 4 to acquire a percentage score of 0–100, with 0 representing the worst possible and 100 denoting the best possible quality of life. In a study examining the clinical validity of the scale [31], the WHO-5 was reported to have a Cronbach’s alpha value of 0.858, indicating good internal consistency. Similarly, this study showed the high internal consistency of the scale, with a Cronbach’s alpha value of 0.872.

#### 2.2.4. Resilience

The 2-item Connor–Davidson Resilience Scale (CD-RISC2), designed by Connor et al. [32], consists of a single scale containing two items used to measure individual resilience. Participants self-rated their resilience on a 5-point Likert scale, from 1 (not true at all) to 5 (always true). The scores of the two items are consequently summed to provide a total resilience score ranging from 2 to 10, with a higher score indicating greater resilience. The CD-RISC2 has shown good test–retest reliability and validity [32]. This study’s Cronbach’s alpha value is 0.836, indicating an acceptable level of internal consistency.

#### 2.2.5. Depressive Symptoms

The Center of Epidemiological Studies Depression Scale for Children (CES-DC) was designed by Weissman et al. [33]. The 20-item CES-DC is used to assess the severity of depressive symptoms experienced within the past week with a 4-point Likert scale. The CES-DC is a widely used measure for screening and monitoring depressive symptoms in children and adolescents [34]. Participants self-rate their perceived frequency of symptoms from 0 (not at all) to 3 (a lot), with higher ratings typically signifying a greater degree of depression symptoms (apart from items 4, 8, 12, and 16, which are reverse-scored). The scores of the 20 items are summed, resulting in a composite score ranging from 0 to 60 that represents the severity of depressive symptoms in the individual. A score greater than 15 indicates a risk of depression in children and adolescents. The CES-DC has displayed a high internal consistency (α = 0.89) [34]. Likewise, in this study, the Cronbach’s alpha value of the scale is 0.914, supporting a strong internal consistency.

### 2.3. Data Analysis

Descriptive statistics were performed for the demographic variables in terms of frequency and percentage and for the study variables, with the mean and standard deviation for the total sample, boys only, and girls only. Listwise, methods were chosen for missing values. Multicollinearity for each independent variable was tested using tolerance (T) and the variance inflation factor (VIF). The results of T for all independent variables ranged from 0.454 to 0.904 (>0.01), and the values of VIF ranged from 2.203 to 1.106 (<5), both indicating no multicollinearity. Multiple linear regression models were performed to examine the extent to which social–emotional learning, resilience, psychological well-being, and depressive symptom (predictor/independent variables) were associated with physical activity (dependent variables) for the total sample, boys only, and girls only. Subsequently, standardized regression coefficients (β) of independent variables were analyzed to examine the relative importance of each independent variable predicting the dependent variable for the total sample, boys only, and girls only. All statistical analyses were performed using SPSS statistical software (version 29.1, SPSS Inc., Chicago, IL, USA) with a significant level set at *p* ≤ 0.05).

## 3. Results

### 3.1. Descriptive Statistics

Table 2 presents the descriptive statistics of the study variables for the total sample and by gender. As shown in Table 2, for the total sample, the mean score of SEL was 4.08 out of a 5-point Likert scale, which portrays an elevated competency of social–emotional learning [28]. The mean score of resilience was 3.99 out of a 5-point Likert scale, showing above-average resilience in the participants. The mean score of PWB was 4.61 out of 5, which indicates a high level of positive well-being. Lastly, the depression mean score was 1.31 out of 4, reflecting a low frequency of depressive symptoms and good mental health. For females, the mean scores of SEL, resilience, psychological well-being, and depression were 4.21, 3.99, 4.65, and 1.34, showing an elevated competency of sociality and resilience, along with robust mental health and uncommon depressive symptoms. For males, the mean scores of SEL, resilience, psychological well-being, and depression were 3.99, 4.02, 4.59, and 1.28, showing similar outcomes to the female sample. Table 3 presents the results of the multiple linear regression models, which examine how associative SEL, resilience, PWB, and depressive symptoms are with the total PA levels within the total sample, male group, and female group.

### 3.2. Association of Perceived SEL, RES, PWB, and DEP with PA in Total Sample

For the total sample, the results of the regression model indicated that SEL, resilience, PWB, and depressive symptoms were significantly associated with the total weekly PA (F = 26.937, *p* < 0.001), explaining 17.2% of the variance in the independent variables (Table 3). Subsequently, among the predictors, PWB was the most individually significant predictor of the total weekly PA (β = 0.383, *p* < 0.001), indicating its strongest association with the outcome variables. Resilience was the second strongest significant predictor of the total weekly PA (β = 0.146, *p* < 0.01). The other variables were not individual significant predictors of total PA (Table 3). Table 4 presents the complete correlation values between variables regarding the total sample.

### 3.3. Association of Perceived SEL, RES, PWB, and DEP with PA in Male Sample

For the male sample, the regression model for all factors collectively was also significant in predicting physical activity levels (F = 15.846, *p* < 0.001), explaining 19.1% of the variance in the independent variables (Table 3). In this sample, PWB was the strongest significant individual predictor of the total weekly PA (β = 0.358, *p* < 0.001), reflecting its prominent role as a predictor (Table 3). Resilience also showed a significant positive association (β = 0.171, *p* = 0.013) with the total weekly PA. SEL and depressive symptoms were not significant individual contributors to predicting the total weekly PA. SEL proved to be the least significant predictor, with a β value of −0.019 and *p* value of 0.800. Table 5 presents the complete correlation values between variables regarding the male sample.

### 3.4. Association of Perceived SEL, RES, PWB, and DEP with PA in Female Sample

For the female participants, the regression model for all factors collectively was also significant in predicting physical activity levels (F = 6.869, *p* < 0.001), explaining 10.6% of the variance (Table 3). Again, PWB was the most negatively significant predictor of the total weekly PA (β = −0.355, *p* < 0.001), while the other variables did not reach statistical significance (Table 3). Resilience emerged to be the least significant predictor (β = −0.060, *p* < 0.471), followed by SEL (β = −0.074, *p* < 0.448), then depressive dymptoms (β = 0.063, *p* < 0.360). Table 6 presents the complete correlation values between variables with regard to the female sample.

## 4. Discussion

The findings from this study underscore the critical role of psychosocial factors as predictors of physical activity among school-aged children. The significant associations observed between these factors and physical activity levels indicate that enhancing these psychosocial factors can lead to increased physical activity.

### 4.1. Total Sample

This study aims to investigate the associations between various psychosocial factors and total weekly physical activity levels among school-aged children. All factors—social–emotional learning, resilience, psychological well-being, and depressive symptoms—contributed to the association drawn with total PA levels when taken together. Of these factors, when considered individually, resilience and psychological well-being and resilience were positively associated with total physical activity levels, while social–emotional learning (SEL) and depressive symptoms did not have strong enough associations to warrant any statistical significance for the total sample of children.

As hypothesized, our findings confirmed the association between resilience levels and the total amount of physical activity among children. The association demonstrated in our study aligns with previous investigations that also suggested a link between higher resilience and increased physical activity in children [15,16,17].

Resilience, as a multifaceted construct, encompasses the capacity to rebound from stress or adversity, fostering a proactive attitude towards life and its challenges [35]. In the context of children’s physical activity, this trait could facilitate the ability to overcome various exercises and their associated challenges, which could include motivation, perceived competencies, and confidence [36]. Children who do not see the various challenges of physical activity as roadblocks, but rather as surmountable obstacles—such as the competitive nature of, and obstacles met during, sport participation—are likely to have higher resilience scores [37], and could be more likely to participate in physical activity as compared to their counterparts. This shift in perspective may be vital in not only supporting children’s persistence in the face of adversity, but also in promoting continued and consistent participation in physical activity. Resilience may also promote a positive feedback loop, in which an initial bout of physical activity spurred by resilience results in feelings of accomplishment and satisfaction. These positive experiences have the power to further strengthen resilience and establish a sustained connection between physical activity and psychological resilience.

As expected, our study found a significant positive correlation between higher psychological well-being scores and levels of physical activity among school-aged children. The association between psychological well-being and physical activity levels is a vital area of research within the realm of public health and psychological research. This association is in accordance with previous publications. Frequency of activity was positively correlated with well-being in the previous literature [38], indicating that this relationship may be consistent across different age groups and cultural contexts. A study further supports these findings, illustrating that in the short term, physical activity promotes happiness and vice versa [21]. This bidirectional relationship suggests that not only does PWB influence physical activity, but engaging in physical activity itself can enhance a child’s mood and mental state.

Despite our previously confirmed associations regarding resilience and psychological well-being on physical activity levels, SEL and depressive symptoms are not significant individual contributors to the total weekly PA for the participants. This finding is generally not aligned with the preconceived relationship between SEL and physical fitness [12,39].

The mechanism of influence regarding the association between SEL and physical activity is likely deeply rooted in common psychological concepts, such as self-efficacy, motivation, and emotional regulation [40], which are all often enhanced by both good mental health and consistent, regular physical activity [41,42,43]. Self-efficacy, or one’s perceived capacity to accomplish certain behaviors, can have a particularly strong influence in children [44]. Children who feel capable of participating in certain physical activities are much more likely to engage in them, which in turn boosts their confidence [45]. With raised confidence will come higher self-efficacy, and therefore further participation in physical activity. Motivation, an intrinsic factor, works in a similar mechanism. Children with a higher motivation for physical activity will want to participate in physical activity, as opposed to other children who may lack such motivation [40,46]. Emotional regulation—the ability to manage and respond to an emotional experience in an adaptive way—has been shown to be positively associated with physical activity [35,47,48], which can act as an outlet for some children to let off unnecessary energy or stress, which in turn could possibly enhance SEL competencies.

Several factors could contribute to the discrepancy between the previous literature and our current study’s findings, shedding light on the potentially complex interplay between emotional intelligence, social skills, and total physical activity engagement [8]. The age range and development stage of participants in our study could influence the observed relationship between SEL and total physical activity level. Our participants are still very young, and significant jumps in social–emotional learning may take time to evolve and shape them [49]. The impact of SEL is subject to change as children grow, as are the motivational factors driving physical activity. This developmental stage that the participants find themselves in could be another possible reason that there is a lack of association between SEL and physical activity level in the current study.

Similar notions should be brought up regarding the association between depressive symptoms and physical activity. There is substantial evidence for physical activity being a predictor of depressive symptoms. Recent meta-analyses, reviews, and trials regarding children’s physical activity and depression have drawn physical activity as being associated with decreased concurrent depressive symptoms [23,50].

This divergence in findings might also be explained by the complex nature of depression in children, which can manifest differently than as seen in adults [51] and may not always inhibit physical activity in straightforward ways [52]. Children may express increased restlessness and agitation, rather than the symptom of lethargy that is commonly attached to adult depression, which could serve as a potential reason why physical activity levels remain relatively stable regardless of depressive symptoms [53,54]. Additionally, younger children are often driven by different motivators than adults [55,56,57]. In this regard, these young children may not yet exhibit the decrease in physical activity typically associated with depressive symptoms, considering their play opportunities are more regulated by opportunity as opposed to motivation.

### 4.2. Gender Differences

Our analysis revealed that PWB was a significant predictor of physical activity levels in both female and male participants, with a slightly stronger association in the male participants. In the male subgroup, PWB displayed a beta coefficient of 0.358, indicating a strong positive association with physical activity levels. This suggests that a higher PWB substantially contributes to an increased PA levels among boys, possibly reflecting the societal norms that encourage physical expression as an aspect of male identity [58,59]. Although the majority of the previous literature regarding this trend is associated with adult males, this potentially extrapolates the trend to younger boys, although further research is likely required to definitively confirm this reasoning. Interestingly, in the female subgroup, a significant negative beta coefficient of −0.355 indicates that as PWB increases, physical activity levels unexpectedly decrease. This inverse relationship is quite intriguing and suggests that for girls, higher levels of reported well-being might be associated with less engagement in physical activity. Girls with a higher PWB might engage in different types of activities that are less physically demanding but equally fulfilling. Social dynamics and peer influence can also play a significant role [60,61]. Girls with a higher PWB might prioritize social interactions that occur in less physically active settings, especially during the sensitive developmental stages covered in school-aged populations.

The role of resilience in influencing physical activity also differed between genders [62]. Among males, resilience showed a significant positive relationship, while in females, resilience did not emerge as a significant predictor. Despite the very limited literature regarding this trend, these findings do align with a previous study regarding gender differences in resilience and physical activity during the COVID-19 pandemic. In this study, females demonstrated a lower resiliency score than the male participants, while perceived stress/resiliency scores were more strongly correlated with physical activity in males [63].

The observed gender differences in the relationship between resilience and physical activity are indicative of potentially distinct coping strategies employed by males and females in response to stress and adversity [64,65,66]. These differences are possibly subject to the differences in socialization experienced by both males and females at a younger age, leading to potentially different methods of handling stress [67]. In the context of physical activity, males are more likely to take up exercise as a form of resilience-building, potentially due to how physical prowess and strength are emphasized in male socialization. On the other hand, female resilience may be supported or manifested through other means, which may not necessarily involve physical activity.

Overall social–emotional learning was not a significant predictor of physical activity in either gender. This finding, in regard to the fact that our analysis did not yield a difference between genders, aligns with previous research [68,69]; however, this finding does challenge some assumptions about the direct impact of SEL on physical activity, as previously mentioned when discussing the total sample.

Finally, depressive symptoms did not significantly predict physical activity in either gender. This finding contrasts with much of the literature suggesting a negative correlation between depression and physical activity, most notable among females [70,71]. The supportive school environments from which our sample was drawn may provide sufficient encouragement and opportunities for physical activity that counterbalance the depressive symptoms that usually deter physical activity [72,73]. This could be another possible insight into the relatively stable levels of depressive symptoms that were demonstrated with altered levels of PA.

### 4.3. Conclusions

This study demonstrates the significant associations between psychosocial factors and physical activity among school-aged children. Our findings highlight that when taken together, social–emotional learning, resilience, psychological well-being, and depressive symptoms are significantly associated with physical activity levels for the total sample, boys, and girls. Individually, psychological well-being and resilience were positively correlated with increased levels of physical activity. The gender-specific analyses reveal nuanced differences, with psychological well-being being a stronger predictor for boys, while resilience was not a significant factor for girls. Based on these findings, we conclude that tailored interventions should be developed that consider the distinct needs and motivations of different genders and children in general to effectively promote PA and overall mental health. The practical implications of our study suggest that educators and policymakers should integrate psychosocial factors into physical education curricula as a means to enhance engagement in PA, potentially reducing the risk of obesity and improving mental health outcomes. Scientifically, the current study contributes to the growing body of literature on the interplay between psychosocial factors and PA, particularly highlighting the importance of PWB and resilience in promoting PA among children.

### 4.4. Limitations

The current study has limitations that should be of note when considering its findings. Firstly, the study’s cross-sectional design limits the ability to infer causality between the various psychosocial variables and physical activity. The self-reported measures used to track variables in the study may be subject to bias. Considering SEL, it is important to discuss the specificity of the SEL scale that we used in the current study. Our SEL scale categorized all of social–emotional learning into 5 overarching categories: self-awareness, self-management, social awareness, relationships, and decision-making. The categorization of our social–emotional learning survey could have been too specific in regard to the questions being asked, which could potentially serve as a reason as to why there was not a significant association drawn between SEL and physical activity. Future research could benefit from a more nuanced exploration of SEL components, to determine which specific skills or subcategories most strongly predict physical activity engagement. Finally, the data collection was conducted two weeks prior to the implementation of the larger study’s intervention. This timing might have influenced the participants’ responses, especially if they had preconceptions of the upcoming intervention. The current study did not find a significant association between depressive symptoms and physical activity, contrary to other current literature. This could be attributed to the nature of the statistical analysis and experimental setup of the study. The current study aimed to identify depressive symptoms as a potential predictor of physical activity levels, which contrasts with most of the previous literature, which takes physical activity levels as a predictor for depressive symptoms. Moreover, the sample size may not have been sufficient to detect a significant association between depressive symptoms and PA, especially when considering potential confounding factors like socioeconomic status or environmental influences.

### 4.5. Future Directions

Future research should firstly consider implementing a longitudinal study design, tracking children as they age and as their psychosocial factors change over time with physical activity. This will help clarify the directionality of the relationships between variables. Objective measures of physical activity should also be implemented in future studies. Self-reporting is subject to bias; therefore, objective measures that can accurately track physical activity will increase the validity of the findings. The current study also focuses on only a subset of psychosocial variables. Researchers should consider broadening the range to include factors such as anxiety, body image, and peer influence when considering physical activity, especially for children.

## Figures and Tables

**Table 1:** Participant demographics.

Variable	Frequency	Percentage
Sex		
Male	281	52.6
Female	240	44.9
Other	13	2.4
Age		
8	1	0.2
9	169	31.6
10	236	44.2
11	78	14.6
12	49	9.2
13	1	0.2
Grade		
4th	415	77.7
6th	119	22.3
Race		
American Indian or Alaska Native	11	2.1
Asian	4	0.7
Black or African American	50	9.4
Native Hawaiian or other Pacific Islander	13	2.4
White	456	85.4

**Table 2:** Descriptive statistics of the variables for the total sample and by gender.

Variables	Total (*n* = 523)	Male (*n* = 279)	Female (*n* = 237)	
	Mean (SD)	Mean (SD)	Mean (SD)	Mean Gender Difference
PA Total	2.83 (1.54)	2.917 (1.51)	2.75 (1.47)	0.167
SEL	4.08 (1.58)	3.99 (1.44)	4.21 (1.40)	−0.22
RES	3.99 (1.99)	4.02 (1.99)	3.99 (1.83)	0.03
PWB	4.61 (2.51)	4.59 (2.35)	4.65 (2.55)	−0.06
DEP	1.31 (1.55)	1.28 (1.53)	1.34 (1.56)	−0.06

**Table 3:** Multiple linear regression models with perceived influence of physical activity on social–emotional learning, resilience, psychological well-being, and depression.

Variable		R	R^2^	F	*p*	df	β	t	*p*
Total									
	Model	0.415	0.172	26.937	<0.001	(0.522)			
	SEL						−0.070	−1.180	0.239
	RES						0.146	2.806	0.005 **
	PWB						0.383	6.855	0.001 **
	DEP						0.064	1.521	0.129
Male									
	Model	0.437	0.191	15.846	<0.001	(0.272)			
	SEL						−0.019	−0.254	0.800
	RES						0.171	2.505	0.013 *
	PWB						0.358	4.997	0.001 **
	DEP						0.062	1.072	0.285
Female									
	Model	0.325	0.106	6.869	<0.001	(0.236)			
	SEL						−0.074	−0.760	0.448
	RES						−0.060	−0.722	0.471
	PWB						−0.355	3.916	0.001 **
	DEP						0.063	0.917	0.360

Note: SEL: social–emotional learning, RES: resilience, PWB: psychological well-being, DEP: pepressive symptoms; ** indicating *p* < 0.01, * indicating *p* < 0.05.

**Table 4:** Correlations between variables for the total sample.

	PA	SEL	RES	PWB	DEP
PA	-	0.260 **	0.294 **	0.396 **	−0.061
SEL	0.260 **	-	0.614 **	0.674 **	−0.269 **
RES	0.294 **	0.614 **	-	0.537 **	−0.228 **
PWB	0.396 **	0.674 **	0.537 **	-	−0.288 **
DEP	−0.061 **	−0.269 **	−0.228 **	−0.288 **	-

Note: ** indicating *p* < 0.01.

**Table 5:** Correlations between variables in the male sample.

	PA	SEL	RES	PWB	DEP
PA	-	0.282 **	0.313 **	0.412 **	−0.068
SEL	0.282 **	-	0.556 **	0.619 **	−0.255 **
RES	0.313 **	0.556 **	-	0.474 **	−0.276 **
PWB	0.412 **	0.619 **	0.474 **	-	−0.245 **
DEP	−0.68	−0.255 **	−0.276 **	−0.245 **	-

Note: ** indicating *p* < 0.01.

**Table 6:** Correlations between variables in the female sample.

	PA	SEL	RES	PWB	DEP
PA	-	0.189 **	0.201 **	0.314 **	−0.055
SEL	0.189 **	-	0.644 **	0.703 **	−0.390 **
RES	0.201 **	0.644 **	-	0.572 **	−0.227 **
PWB	0.314 **	0.703 **	0.572 **	-	−0.374 **
DEP	−0.055	−0.390 **	−0.227 **	−0.374 **	-

Note: ** indicating *p* < 0.01.

## Data Availability

The original contributions presented in this study are included in the article, and further inquiries can be directed to the corresponding author.
